# Acute Psychosis in the Setting of Undiagnosed Normocalcemic Hyperparathyroidism: A Case Report

**DOI:** 10.7759/cureus.35840

**Published:** 2023-03-06

**Authors:** Ali El-Husari, Davong D Phrathep, Mohamed Ibrahim, Danielle Chism, Ravish Narvel

**Affiliations:** 1 College of Osteopathic Medicine, Lake Erie College of Osteopathic Medicine, Bradenton, USA; 2 Internal Medicine, Ascension St. Vincent's Riverside, Jacksonville, USA

**Keywords:** acute kidney injury, acute hypercalcemia, electrolytes abnormalities, psychosis, normocalcemic hyperparathyroidism

## Abstract

Hyperparathyroidism (HPT) causes an elevation of parathyroid hormone (PTH). This can result in elevated calcium levels, which can cause bone, kidney, muscle, gastrointestinal, and neuropsychiatric symptoms, including psychosis. Our report presents a unique case of an elderly woman who presented to the emergency department in an unconscious state with a working diagnosis of metabolic encephalopathy secondary to sepsis and urinary tract infection. Despite fluids and antibiotic treatment, the patient showed hallucinations. The acuteness of her psychotic episodes prompted the medical team to further investigate the cause of her hallucinations. Additional labs revealed HPT, which she had never been diagnosed with prior to the hospital admission. Our patient’s novel clinical presentation revealed elevated PTH and normal calcium levels as the cause of her psychosis. We determined that the normal calcium levels were due to the patient’s calcium loss secondary to acute kidney injury. Cinacalcet administration showed resolution of the patients' hallucinations, highlighting the importance of PTH screening even in normocalcemic patients. In this report, we present a rare clinical presentation of acute psychosis in the setting of undiagnosed normocalcemic HPT.

## Introduction

Hyperparathyroidism (HPT) is a condition where serum levels of the parathyroid hormone (PTH) are elevated. HPT can be primary, due to excess PTH production mostly from the parathyroid glands themselves, or secondary, due to decreased serum calcium, which increases levels of PTH. PTH increases calcium levels, which eventually leads to the classical symptoms of HPT, nephrolithiasis, and severe bone disease. Patients with HPT today usually lack the classic symptoms, due to early intervention, but have nonspecific complaints such as fatigue, decreased concentration, sleep problems, bone or muscle pains, and gastroesophageal reflux disease [1]. HPT can also cause neuropsychiatric issues ranging from depression and memory loss to thoughts of death and suicide or even hallucinations. According to studies cited by Parks et al. [2], 5-20% of patients who are undergoing a parathyroidectomy due to HPT have symptoms of delusions and hallucinations.

The pathophysiology behind hallucinations from HPT has remained unclear. There is no clear correlation between the degree of hypercalcemia and the severity of symptoms [3]. Neuropsychiatric symptoms have also been seen in patients with normocalcemic HPT [4].

Normocalcemic HPT is characterized as having normal serum calcium levels with elevated PTH on two separate occasions within six months [4]. It can also be of primary or secondary etiology, from excess glandular production or due to calcium loss in some fashion, including renal disease and bariatric surgery [4]. Rao et al. [5] proposed that HPT is a biphasic process, suggesting that PTH is elevated initially without raising levels of calcium.

Here, we present a case of a 72-year-old female with no psychiatric background, with acute onset psychosis secondary to HPT. This case is novel due to the lack of elevated calcium levels while she still suffered from acute and recurrent symptoms of psychosis.

## Case presentation

A 72-year-old female was admitted to the hospital after falling and being found unconscious at home. Her neighbors allied for a wellness check after not seeing her for nine days. In the hospital, the patient showed recurrent hallucinations. Her past medical history was positive for hypertension, obesity, and diabetes mellitus. The patient reported no past history of seizure disorder, schizophrenia, or psychosis. On physical examination, the patient had multiple large ecchymotic lesions on her back and a large sacral ulcer as well as a heel wound from falling and being on the floor for so long. She was initially hypotensive and tachycardic. Her blood pressure was stabilized after IV hydration with two liters of crystalloids to 127/71. Her heart rate was 121, oxygen saturation was 98%, and the temperature was 37.3°C. Her lab values are shown in Table 1.

**Table 1:** Initial lab values mEq/L: milliequivalents per liter; mg/dL: milligrams per deciliter; g/dL: grams per deciliter; mIU/mL: milli-international units per milliliter; ng/dL: nanograms per deciliter; mmol/L: millimoles per liter; WBC/L: white blood cells per liter.

Laboratory parameter (units)	Patient values	Reference range
Sodium (mEq/L)	155 mEq/L	135-145 mEq/L
Potassium (mEq/L)	3.9 mEq/L	3.5-5 mEq/L
Chloride (mEq/L)	116 mEq/L	96-106 mEq/L
Carbon dioxide (mEq/L)	26 mEq/L	23-29 mEq/L
Glucose (mg/dL)	530 mg/dL	70-100 mg/dL
Anion gap (mEq/L)	13 mEq/L	4-12 mEq/L
Albumin (g/dL)	3.5 g/dL	3.4-5.4 g/dL
Calcium (mg/dL)	12.9 mg/dL	8.5-10.2 mg/dL
Thyroid-stimulating hormone (mIU/mL)	0.27 mIU/mL	.35-4.50 mIU/mL
Free thyroxine (ng/dL)	1.8 ng/dL	0.7-1.9 ng/dL
Ionized calcium (mmol/L)	1.6 mmol/L	1.16-1.31 mmol/L
Blood urea nitrogen (mg/dL)	69 mg/dL	6-24 mg/dL
White blood cells (WBC/L)	17.6 x 10^9 ^WBC/L	4.5-11.0 x 10^9 ^WBC/L
Neutrophils (%)	89%	40-60%

Her leukocytosis along with her urinalysis results was consistent with a urinary tract infection. She was originally started on cefepime (2 g IV q12), 3 L of normal saline, 1 L bolus isotonic fluids, fentanyl for pain, and vancomycin (1 g IV q12).

The initial diagnoses were metabolic encephalopathy, acute kidney injury, sepsis secondary to urinary tract infection, hypernatremia and hypercalcemia secondary to dehydration, and hyperglycemia. Additional fluids and an insulin sliding scale were added to her treatment. Later urine cultures were positive for *Proteus*, and her antibiotics were immediately switched to ceftriaxone 1 g IV daily.

The patient completed her antibiotic regimen over the next five days. Her hypernatremia, hyperglycemia, hypercalcemia, and hypokalemia were also corrected. On the 10th day, the patient had uncomplicated surgical debridement of her sacral ulcer. She still reported visual hallucinations even after recovery from surgery; therefore, psychiatry was consulted. She was treated with olanzapine 5 mg at bedtime and intramuscular every six hours as needed. Despite using olanzapine over the seven days, there was no improvement, as she still experienced sustained hallucinations, prompting an additional medical investigation.

Repeat complete metabolic panel (CMP) revealed normal calcium, and additional labs showed high serum PTH levels at 150 pg/mL (normal range: 10-55 pg/mL). At this moment in time, a thyroid ultrasound was ordered. Thyroid ultrasound revealed a 3.8 cm Thyroid Imaging Reporting and Data System (TIRADS) grade 3 nodule in the left lobe of the thyroid gland with no evidence of parathyroid adenoma. This ruled out a parathyroid tumor as the cause of the patient’s symptoms. The patient's normal calcium levels were attributed to the acute kidney injuries she had sustained. The patient was started on cinacalcet 300 mg twice a day for the diagnosis of primary HPT. After two weeks on cinacalcet, the patient reported the resolution of her hallucinations. Repeat calcium and PTH levels were unremarkable. The resolution of the patient’s hallucinations after cinacalcet treatment revealed that the cause of her acute psychosis was primary HPT.

## Discussion

HPT is characterized by hypercalcemia and elevated PTH. Although rare, it can present with acute psychosis as well as confusion, paranoia, disorientation, and hallucination. The first collection of HPT cases presenting with psychiatric symptoms was reported by Eitinger in 1942. It described seven out of the 50 patients exhibiting mental symptoms such as confusional psychosis, drowsiness, and stupor [6]. Similarly, a clinical analysis and literature review by Karpati and Frame in 1964 described 33 cases of HPT, in which 14 of the cases (42%) reported psychiatric or neuromuscular symptoms [7]. Four of those 14 cases showed a predominance of psychotic features with marked improvement only after the reversal of the hormonal imbalance [7]. Karparti and Frame described several instances of personality changes, psychoneurotic symptoms, and even overt psychosis with delusions and hallucinations in their assessment of the literature [7]. A review by Agras and Oliveau found the incidence of psychiatric symptoms to be 4.2% in 405 cases of HPT [6]. Although the incidence is rare, HPT with psychiatric symptoms has been well-documented for decades.

The exact mechanism behind HPT-induced psychosis remains ill-defined and unknown. A study by Brown et al. hypothesized that the high extracellular calcium concentration triggered a downward influx into neurons, causing depolarization [8]. This abnormal signaling within neurons and increased release of neurotransmitters may be responsible for the psychiatric symptoms since calcium-channel blockers are used as adjunct therapy in the treatment of mania and other psychiatric conditions [8]. A study by Petersen also described the serum calcium levels correlated with the severity of mental disturbances, i.e., the higher the calcium levels, the more severe the mental disturbance [9]. However, previous studies [10] suggest that no correlation exists, in which even mild or moderate increases in serum calcium have the potential to precipitate psychiatric symptoms, including psychosis. In 2016, a case report and literature review by Park and Hieber reported several cases of acute psychiatric symptoms associated with hypercalcemia and HPT, which showed the reversibility of psychotic symptoms with either parathyroidectomy or corrected serum calcium levels [11]. Although the pathophysiology behind psychosis in patients with HPT is undefined, cases continue to be reported.

An interesting and unusual phenomenon associated with our patient was that she initially presented with hypercalcemia that was corrected with fluids, but her PTH levels remained elevated at 150 pg/ml, indicating a diagnosis of normocalcemic HPT. Normocalcemic HPT can be due to primary causes such as an adenoma, multinodular goiter, or just hyperplasia. It can also be due to secondary causes such as vitamin D deficiency, renal failure, or intestinal malabsorption of vitamin D or calcium [4]. With a blood urea nitrogen (BUN) of 69 mg/dL and prolonged immobilization, an acute kidney injury of prerenal azotemia and potential rhabdomyolysis from muscle breakdown could have caused an acute drop in calcium, masking a diagnosis of primary HPT [12]. The mechanism would be due to acute kidney injury during rhabdomyolysis causing phosphate retention due to impaired phosphate use in the muscles as well as a lack of excretion by the kidneys [12]. The inability of the muscles to use phosphate through the adenosine triphosphate (ATP) cycle causes a depletion in ATP, and ultimately impairs the calcium transport pumps in the muscles [12]. The rise in phosphate exacerbates the deposition of calcium phosphate in damaged muscles and other tissues, giving rise to acute hypocalcemia in the presence of acute kidney injury and potential rhabdomyolysis [12].

With regard to workup and management, this case report describes a unique scenario in which the calcium levels were normocalcemic due to acute kidney injury masking the effects of HPT. The unknown mechanism of HPT-induced psychosis continues to be a gap in knowledge that could help assess which patient profiles are at higher risk for psychiatric symptomatology. Although the pathophysiology is unknown, the current treatment for patients with acute psychosis due to HPT is well-defined. Treatment for acute psychosis due to alterations in calcium levels is to treat the underlying cause rather than administering psychiatric medication [13]. In addition to treatment involving correction of calcium levels, other cases have shown that parathyroidectomy is curative [1-2,4,13]. As cases of acute psychosis continue to be reported, there is a need for more studies to be conducted on the mechanisms of how psychosis is induced by normocalcemic HPT. A retrospective cohort study could be conducted to assess which patients with HPT may have a higher risk of psychosis, such as certain acute issues like acute kidney injury. Such studies will allow providers to identify high-risk patients and anticipate the need for specialized care to treat underlying disruption in calcium levels for this patient group.

## Conclusions

HPT should be included in the differential diagnosis of elderly patients who suffer from neuropsychiatric symptoms since correction could result in remission. Normal electrolytes cannot be used to rule out HPT since renal injury can result in altered levels. At the time of this writing, there is limited literature that discusses acute psychosis associated with HPT in a normocalcemic patient due to renal injury or otherwise. Therefore, we provide a unique case report of acute psychosis in the setting of undiagnosed normocalcemic HPT. We urge more research to be completed on the mechanism involving normocalcemia, and HPT as the cause of rapid-onset hallucinations in elderly patients.
